# Impact of Parental Time-Restricted Feeding on Offspring Metabolic Phenotypic Traits

**DOI:** 10.7150/ijbs.107469

**Published:** 2025-02-10

**Authors:** Yibo Fan, Xiangyuan Peng, Nishat I. Tabassum, Xiangru Cheng, Sharmelee Selvaraji, Vivian Tran, Tayla A. Gibson Hughes, Buddhila Wickramasinghe, Abdulsatar Jamal, Quynh Nhu Dinh, Mathias Gelderblom, Grant R. Drummond, Christopher G. Sobey, Jim Penman, Terrance G. Johns, Raghu Vemuganti, Jayantha Gunaratne, Mark P. Mattson, Dong-Gyu Jo, Maria Jelinic, Thiruma V. Arumugam

**Affiliations:** 1 Centre for Cardiovascular Biology and Disease Research, La Trobe Institute for Molecular Science, La Trobe University, Melbourne, Australia.; 2 Department of Microbiology, Anatomy, Physiology and Pharmacology, School of Agriculture, Biomedicine and Environment, La Trobe University, Melbourne, Australia.; 3 Research Laboratory of Electronics, Department of Materials Science and Engineering, Massachusetts Institute of Technology, Boston, MA, USA.; 4 Department of Neurology, University Medical Center Hamburg-Eppendorf, Hamburg, Germany.; 5 Epigenes Australia Pty Ltd., Melbourne, Victoria, Australia.; 6 Department of Neurological Surgery, University of Wisconsin, Madison, WI, USA.; 7 Translational Biomedical Proteomics Laboratory, Institute of Molecular and Cell Biology, Agency for Science, Technology and Research, Singapore, Singapore.; 8 Department of Anatomy, Yong Loo Lin School of Medicine, National University of Singapore, Singapore, Singapore.; 9 Department of Neuroscience, Johns Hopkins University School of Medicine, Baltimore, MD, USA.; 10 School of Pharmacy, Sungkyunkwan University, Suwon, Republic of Korea.

**Keywords:** Intermittent fasting, Metabolic Syndrome, Intergenerational inheritance

## Abstract

Intermittent fasting (IF) is widely recognized for its numerous health benefits, yet its impact on metabolic health across generations remains relatively unexplored. This study investigates the intergenerational effects of parental IF, specifically through 8-hour daily time-restricted feeding, on the metabolic health of offspring. By examining four different combinations of parental mating groups, we demonstrate that parental IF can influence offspring metabolic health in distinct ways. Our results reveal that parental IF conferred significant metabolic advantages compared to ad libitum (AL) feeding. IF parents exhibited lower glucose, HbA1c, cholesterol, and CRP levels, and higher ketone levels compared to AL parents. Offspring of IF-exposed animals displayed sex-specific metabolic benefits when challenged with a high-fat, high-sugar, and high-salt (HFSS) diet. Notably, female offspring from IF parents were protected against HFSS-induced glucose intolerance and exhibited lower plasma glucose levels and higher ketone levels compared to offspring of ad libitum-fed parents. Additionally, female offspring from IF parents on a HFSS diet, along with both female and male offspring on a normal diet, had elevated plasma insulin levels. Furthermore, male offspring from IF parents on a normal diet exhibited a significant reduction in body weight compared to offspring from AL parents. These findings suggest that parental IF can impart enduring metabolic benefits to offspring and may serve as an effective strategy to mitigate the risks of obesity and diabetes in future generations.

## Introduction

Over the past nine decades, a multitude of studies exploring the effects of dietary restriction, encompassing caloric restriction (CR) and intermittent fasting (IF), have consistently revealed their ability to enhance metabolic health and confer resistance to a range of chronic diseases. IF, which involves alternating periods of fasting and unrestricted eating while maintaining nutritional adequacy, has been shown to mitigate the development of age-related cardiovascular, neurodegenerative, and metabolic disorders, and to promote increased longevity 1-4. CR and IF not only offer protective effects against age-related ailments but also exhibit positive impacts on cognition and cellular resilience against various environmental challenges.

Epigenetic modifications are critical in mediating how environmental factors influence genomic regulation. Many age-related diseases are polygenic and influenced by environmental conditions, suggesting that the interaction between environmental factors and genetic predispositions plays a key role in the complex pathophysiology of chronic diseases. These interactions shape gene expression patterns through various epigenetic mechanisms, including DNA methylation, histone modifications, histone remodeling, and microRNA activity. Recent studies have demonstrated that dietary restrictions, including IF, lead to significant epigenetic changes and alterations in gene expression in animal models 5-8. Our research builds on this understanding by investigating how IF impacts the epigenome. Specifically, IF has been shown to alter histone trimethylation, resulting in extensive transcriptomic changes that drive metabolic adaptations 9. Additionally, global DNA methylation patterns in animals subjected to IF exhibit distinct alterations in CG-rich nucleotide islands (CGIs) 10. Despite the well-documented health benefits of IF in both animals and humans 11, the precise nature and extent of IF-induced epigenetic changes, and the prospect of intergenerational inheritance of such changes are unknown.

Previous studies addressing the impact of parental IF on mouse offspring have specifically focused on maternal IF. Studies by Yin and colleagues (2021) examined the effect of alternate-day maternal IF on the DNA methylation status of the liver in offspring and identified a correlation between maternal IF and decreased hepatic global DNA methylation in adult offspring 12. Another study by the same group investigated the effect of long-term pre-pregnancy IF on offspring metabolism and phenotypes. Neonatal offspring from maternal IF weighed significantly less compared to control offspring; however, their adipose tissue mass was significantly increased 13. Maternal IF also significantly decreased levels of DNA methyltransferase in the liver of offspring, and DNA methylation modifications of molecules associated with the mTORC1 signaling pathway were significantly altered, leading to the significant inhibition of mTORC1 signaling 13. Furthermore, that long-term IF before pregnancy disrupts intestinal homeostasis in offspring, leading to subsequent disorders in glucose and lipid metabolism 14. Another study using rats and employing 16-hour daily IF found that maternal IF does not affect resting cardiovascular, metabolic, and renal function; however, when challenged by dietary salt load, male IF offspring are more prone to renal injury 15. While there is some knowledge in the literature about how maternal IF may affect offspring health, there is no information on how combined maternal and paternal IF, as well as the effects of paternal or maternal IF alone, may influence offspring metabolic health.

It is therefore of interest to explore whether IF represents an environmental trigger capable of modulating phenotypic profiles not only in parents but also in their descendants. Therefore, this study aims to explore whether IF acts as an environmental factor capable of shaping not only the metabolic profiles of parents but also those of their descendants. In this study, we investigated the intergenerational effects of IF in one or both parents on the metabolic phenotypes of their offspring.

## Materials and Methods

### Animals and IF procedures

**Figure 1:**
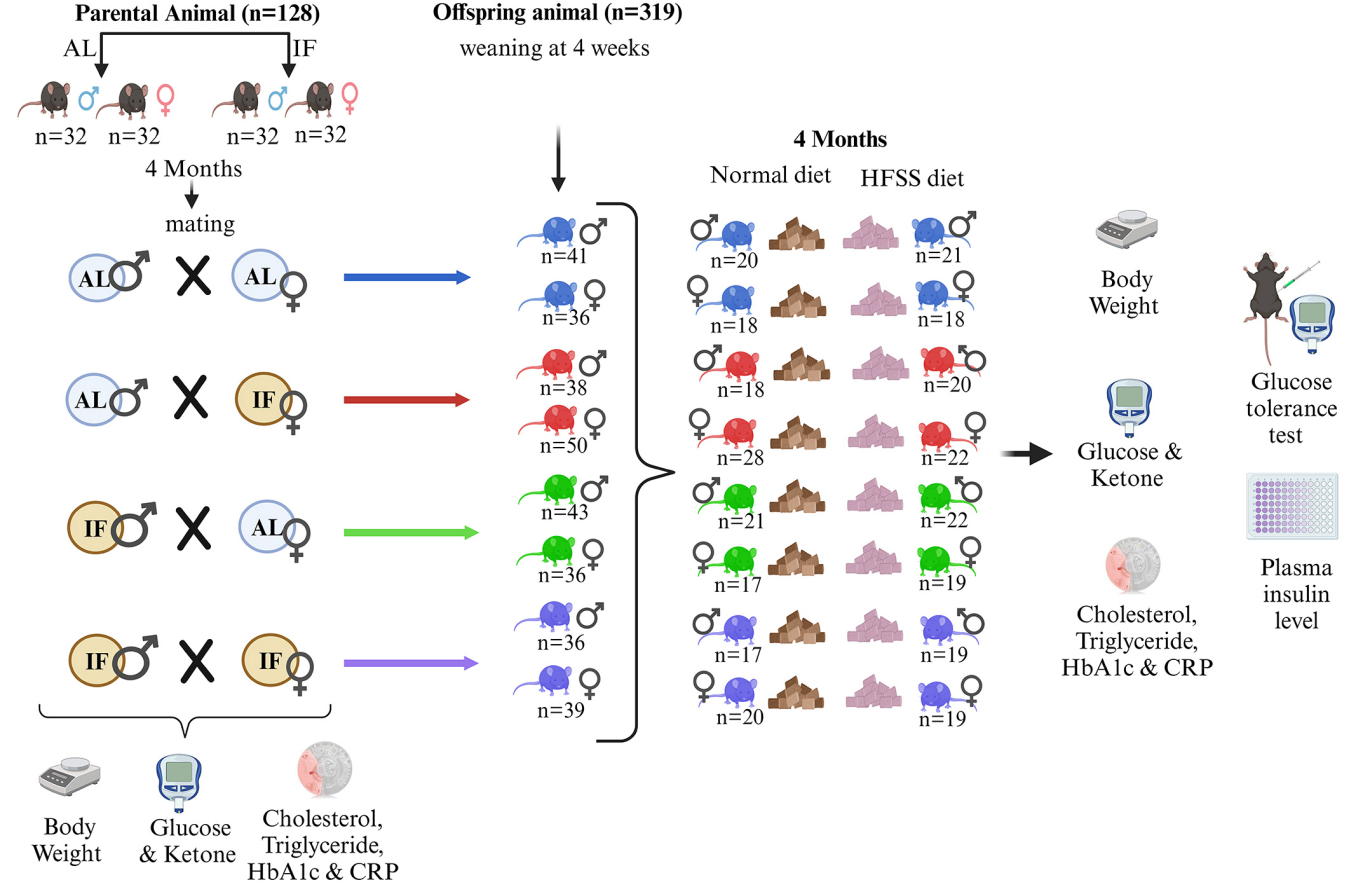
** Experimental Design for Parental Animals and Offspring.** Parental animals were divided into two groups: one received ad libitum (AL) access to food, while the other underwent daily 16-hour intermittent fasting (IF16) for 4 months before mating. All parental mice were raised under AL conditions for 6 weeks before being randomly assigned to either continue AL feeding or undergo IF16. Offspring were separated from their mothers at 4 weeks of age and further divided into male and female groups. These groups were then subdivided into those receiving a normal diet or a high-fat, high-sugar, high-salt (HFSS) diet, and were maintained under these dietary conditions for 16 weeks.

All *in vivo* experimental procedures were approved by La Trobe University (Ethics approval number: AEC21047) Animal Care and Use Committees and conducted in accordance with the guidelines outlined in the Australian Code for the Care and Use of Animals for Scientific Purposes (8th edition) and the NIH Guide for the Care and Use of Laboratory Animals. Every effort was made to minimize suffering and reduce the number of animals used. All sections of the manuscript were performed in accordance with ARRIVE guidelines. C57BL/6 male and female mice were purchased at 4 weeks of age from ARC, Australia, and housed in the animal facility at La Trobe University. The animals were subjected to a 12-hour light:12-hour dark cycle (07:00-19:00) and provided with normal diet comprising 20% total crude protein, 8.5% crude fat, 3.2% crude fiber, 1.1% calcium and 0.96% phosphorus (Barastoc WEHI mouse breeder cubes Irradiated; Ridley, Australia). Water was available ad libitum to all dietary groups. At 6 weeks of age, male and female mice were randomly assigned to different dietary intervention groups: Intermittent Fasting for 16 hours (IF16; n=64) and ad libitum feeding as a control (AL; n=64) (**Fig. 1**). Mice in the IF16 group underwent daily fasting for 16 hours for 4 months (16:00-08:00), while the AL group had continuous access to food pellets. Body weight was regularly monitored, and blood glucose and ketone levels were measured using the FreeStyle Optium Neo system with FreeStyle Optium blood glucose and ketone test strips (Abbott Laboratories, Illinois, USA) 6 hours after fasting (**Fig. 1**). Additionally, cholesterol (Roche Cobas b 101 Lipid, Cat. No. ROC06380115190, Roche Diagnostics, Basel, Switzerland), triglycerides (Roche Cobas b 101 Lipid, Cat. No. ROC06380115190, Roche Diagnostics, Basel, Switzerland), HbA1c (Roche Cobas b 101 HbA1c, Cat. No. ROC08038694190, Roche Diagnostics, Basel, Switzerland) and CRP (Roche Cobas b 101 CRP, Cat. No. ROC08024669190, Roche Diagnostics, Basel, Switzerland) levels were assessed at different time points from all animals after 6 hours fasting (at the onset of dietary interventions, as well as at 8 and 16 weeks post-intervention). Blood glucose, ketone, and lipid measurements were consistently taken at the same time of day across all groups to minimize variability due to circadian rhythms. After 4 months of dietary intervention, animals were assigned to mating groups (**Fig. 1**). To minimize any potential bias in the formation of mating pairs, we randomly selected animals for pairing. We did not consider factors such as weight or glucose levels in the selection process. This random pairing approach helps ensure that the results are not influenced by these variables. After the mating process and subsequent weaning of offspring were completed, all parental animals were euthanized by carbon dioxide (CO2) inhalation between 7 a.m. and noon. Subsequently, all mice were perfused with cold PBS, and tissue samples were collected and snap-frozen in liquid nitrogen and stored at -80 °C until further use.

### Mating and offspring production

After 4 months of dietary intervention, male and female mice were randomly divided into four groups: AL fathers, IF fathers, AL mothers, and IF mothers. The mating groups were assigned as follows: AL-father X AL-mother; AL-father X IF-mother; AL-mother X IF-father; IF-father X IF-mother. The offspring were obtained and divided into male and female groups upon weaning, with 36-50 animals per group (8 groups in total). Furthermore, both female and male offspring were further divided into normal diet groups and high-fat food and high-salt, and high-sugar drinking water (HFSS; High-fat: 20.90% protein, 23.50% total fat, 5.40% crude fiber, 5.40% AD fiber; SF04-001, Specialty Feeds, Australia; High-salt [0.9% Sodium Chloride solution (Cat. No. AHF7124, Baxter Healthcare, Deerfield, IL, USA)]; High-sugar [4.5% glucose and 5.5% fructose] groups, with 17-28 animals per group (16 groups in total). We used the HFSS diet as it has been demonstrated to be a robust model for inducing low-grade inflammation and metabolic syndrome 16.

Please refer to Figure 1 for details on grouping. All offspring were placed on their assigned dietary regimen for a period of 16 weeks. Body weight was weekly measured. Blood glucose and ketone levels were assessed in the morning using the FreeStyle Optium Neo system with FreeStyle Optium blood glucose and ketone test strips before IF animals received food. The fasting conditions, including the duration of fasting and access to water, were identical across all groups to ensure consistency and minimize variability in the results. Additionally, cholesterol, triglycerides, HbA1c and CRP levels were measured: at the start of different dietary interventions, as well as at 8 and 16 weeks after the interventions.

### Glucose tolerance tests and insulin measurement

Glucose tolerance tests were performed following an overnight fasting period of 16±2 hours, with basal blood glucose and plasma insulin samples (t = 0) measured. Mice were administered 2 grams of glucose per kilogram of body weight via intraperitoneal injection using a 20% glucose solution (Cat. No. 49163, Sigma-Aldrich, St. Louis, MO, USA). Blood glucose levels were monitored at 15-, 30-, 60-, and 120-minutes post-glucose challenge. Additional blood samples were obtained in a 0.8 ml microtube with LH Lithium Heparin Separator (Cat. No. 450535 PK, MiniCollect, Greiner Bio-One, Kremsmünster, Austria) for plasma insulin analysis at 30- and 120-minutes post-glucose injection. To prepare plasma samples, blood samples were centrifuged at 10, 000 RPM for 10 minutes at 4°C, followed by transfer of the plasma to tubes. These tubes were then snap-frozen in liquid nitrogen and stored at -80°C for subsequent insulin measurements. Insulin levels were quantified using Mouse Ultrasensitive Insulin ELISA (Cat. No. 80-INSMSU-E01, ALPCO, Salem, NH, USA), with each sample assayed in duplicate following the manufacturer's instructions. The measurement and analysis of all metabolic parameters were conducted collaboratively by our research team. To ensure unbiased results, all researchers were blinded to the group assignments, with the exception of one individual who was responsible for maintaining the experimental setup and group allocation.

### Statistical analysis

**Figure 2:**
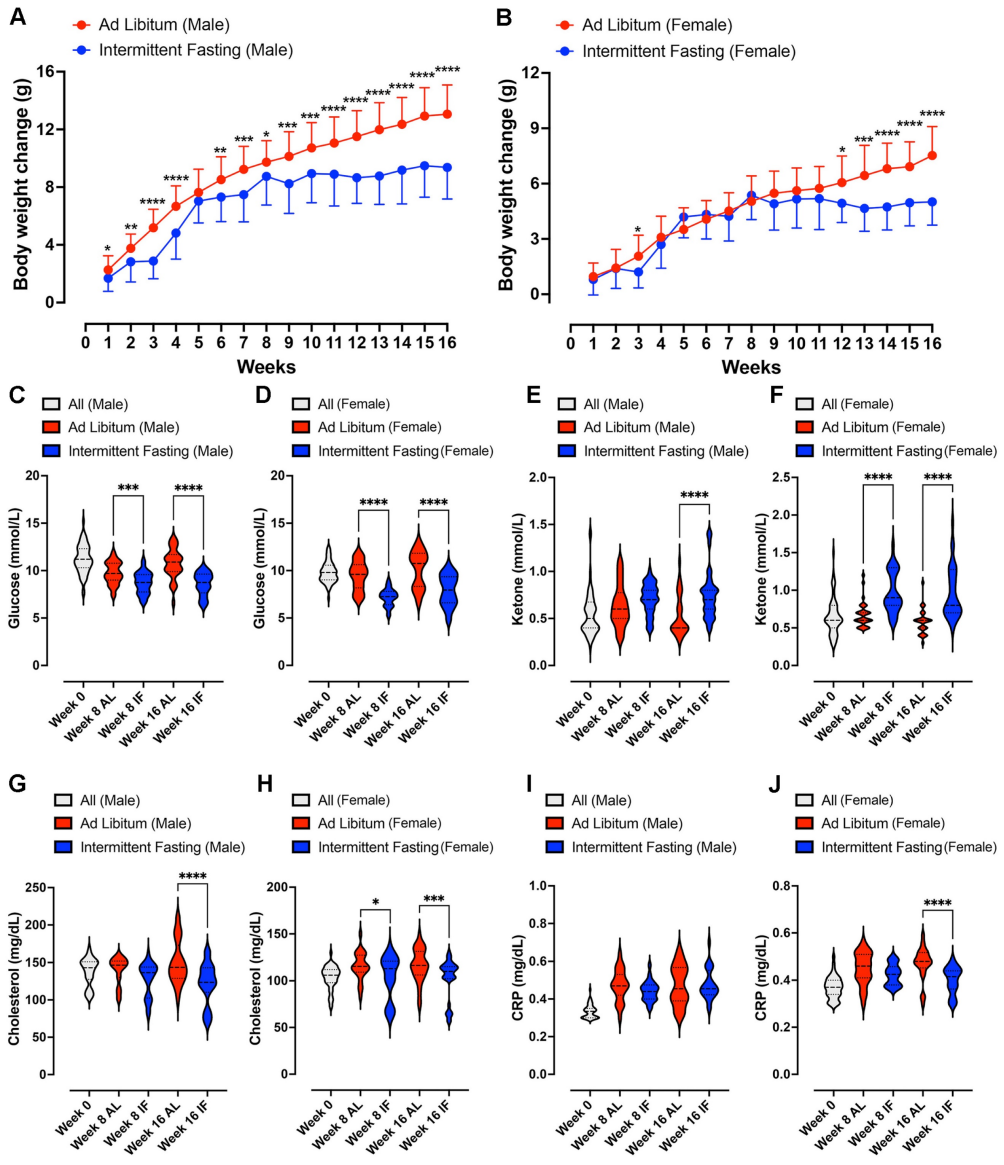
** Impact of Intermittent Fasting on Parental Animals**. (A) and (B) show the weekly body weight changes accumulated compared to the value at week 0 in male and female animals, following daily 16-hour intermittent fasting (IF) over a 16-week period. Data are presented as means ± standard deviation. Statistical analysis was conducted using two-way repeated-measures ANOVA, followed by post-hoc Šídák's multiple comparisons test. Significance levels are denoted as *P < 0.05, **P < 0.01, ***P < 0.001, ****P < 0.0001 compared to IF animals. Each group consisted of n = 32 animals. (C) and (D) display violin plots representing blood glucose levels in male and female animals, respectively. (E) and (F) depict blood ketone levels in male and female animals, while (G) and (H) show cholesterol levels. (I and J) illustrates C-reactive protein levels in male and female parental animals. Statistical analysis for these parameters was conducted using two-way ANOVA, followed by post-hoc Šídák's multiple comparisons test. Significance levels are denoted as *P < 0.05, ***P < 0.001, ****P < 0.0001 compared to *ad libitum* (AL) animals. Each group consisted of n = 32 animals.

**Figure 3:**
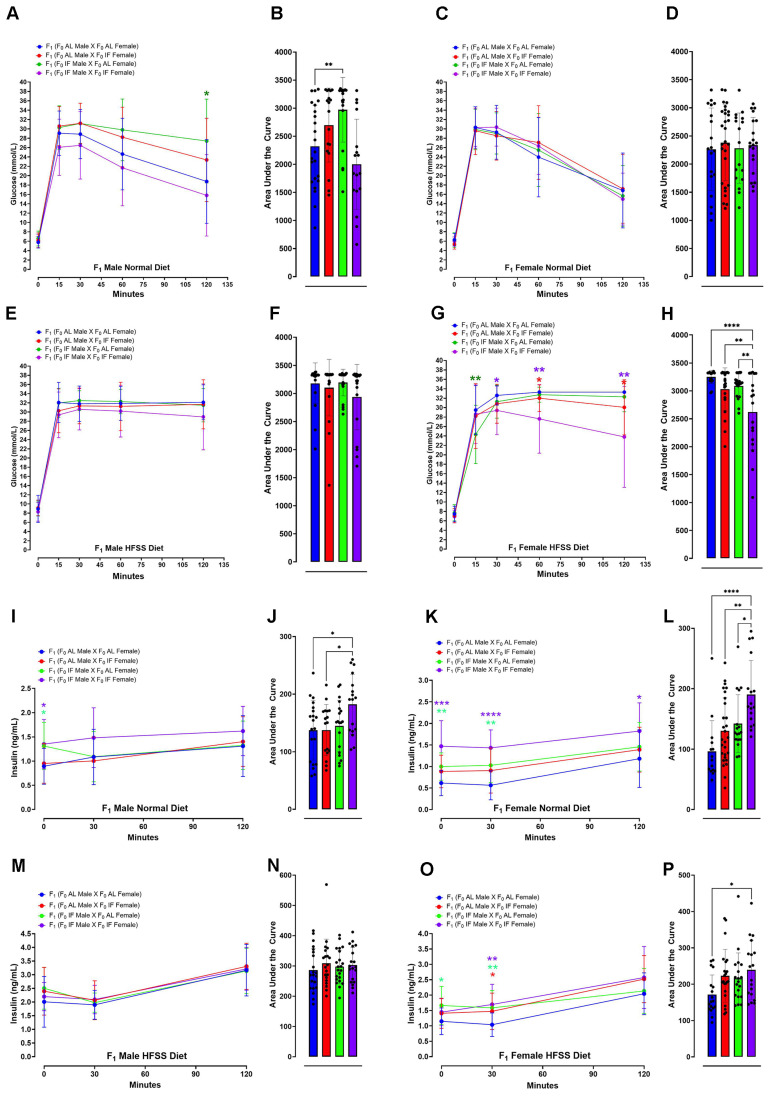
** Glucose and Insulin Response in Offspring Following Glucose Tolerance Test**. Blood glucose concentrations during glucose tolerance test in normal diet male (A and B) and normal diet female (C and D) F_1_ mice at 0, 15, 30, 60 and 120 minutes post intraperitoneal glucose injection. Data are presented as means ± standard deviation. Statistical analysis was conducted using two-way repeated-measure ANOVA, followed by post-hoc uncorrected Fisher's LSD comparisons test. *P < 0.05 versus normal diet F_1_ (F_0_ AL male X F_0_ AL Female) animals. (B and D) Glucose area under the curve (AUC) for normal diet male (B) and normal diet female (D) F_1_ mice. Statistical analysis was performed using one-way ANOVA, followed by Tukey's multiple comparisons test. **P < 0.01, versus F_1_ (F_0_ AL male X F_0_ AL Female) animals. Each group consisted of n = 17-28 animals. Similar analyses were conducted for F_1_ mice fed a HFSS diet. Blood glucose concentrations during glucose tolerance test in HFSS diet male (E and F) and HFSS diet female (G and H) F_1_ mice after intraperitoneal glucose injection. Blood insulin levels during the glucose tolerance test in male (I and J) and female (K and L) F_1_ mice fed a normal diet, measured post intraperitoneal glucose injection. Statistical analysis was conducted using two-way repeated-measure ANOVA, followed by post-hoc uncorrected Fisher's LSD comparisons test. *P < 0.05, **P < 0.01, ***P < 0.001, ****P < 0.0001 denote significance compared to normal diet F_1_ (F_0_ AL male X F_0_ AL Female) animals. Insulin AUC for male (J) and female (L) F_1_ mice fed a normal diet was calculated. Statistical analysis was performed using one-way ANOVA, followed by Tukey's multiple comparisons test. *P < 0.05, **P < 0.01, ****P < 0.0001 indicate significance compared to F_1_ (F_0_ AL male X F_0_ AL Female) animals. Each group consisted of n = 15-28 animals. Similar analyses were conducted for F1 mice fed a HFSS diet. Blood insulin concentrations during the glucose tolerance test in HFSS diet male (M and N) and female (O and P) F_1_ mice were measured at 0, 30, and 120 minutes post injection. Statistical significance was assessed using two-way repeated-measure ANOVA, followed by post-hoc uncorrected Fisher's LSD comparisons test. *P < 0.05, **P < 0.01 denote significance compared to F_1_ (F_0_ AL male X F_0_ AL Female) animals. AUC were determined, with statistical analysis conducted using one-way ANOVA, followed by Tukey's multiple comparisons test. *P < 0.05 denotes significance compared to HFSS F_1_ (F_0_ AL male X F_0_ AL Female) animals. Each group consisted of n = 18-22 animals.

**Figure 4:**
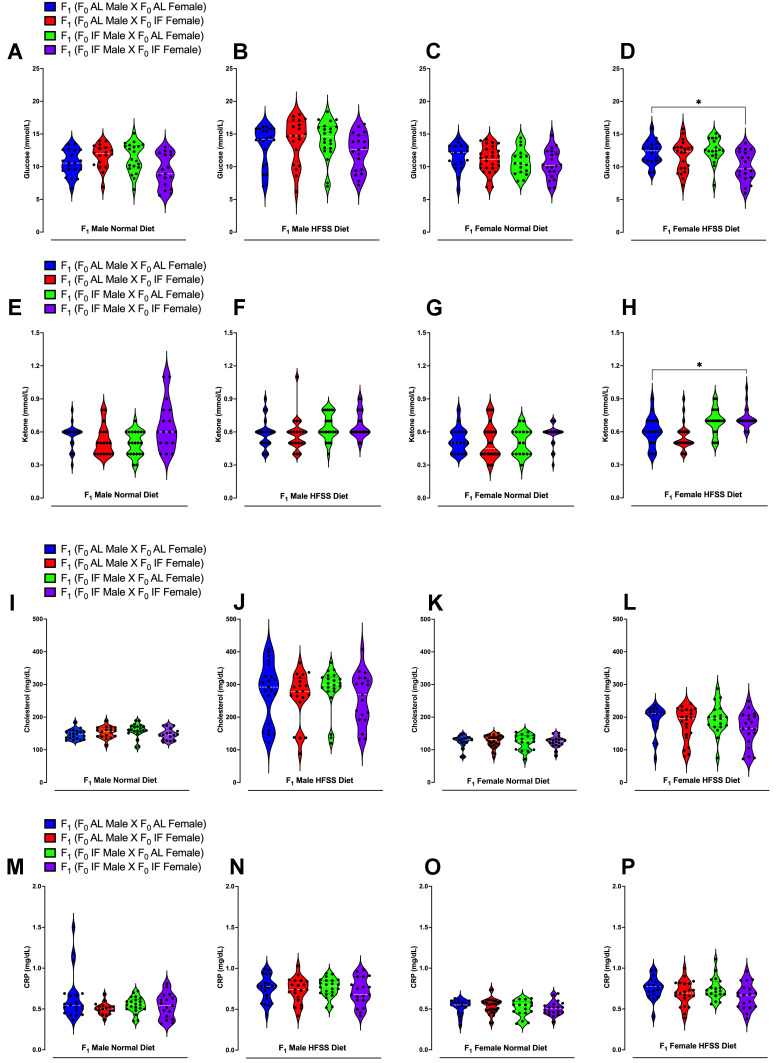
** Blood Glucose, Ketone, Cholesterol and CRP Levels of Offspring Animals.** Blood glucose levels at week 16 in male offspring under normal diet (A), HFSS diet (B), as well as female offspring under normal diet (C) and HFSS diet (D). Blood ketone levels at week 16 in male offspring under normal diet (E), HFSS diet (F), as well as female offspring under normal diet (G) and HFSS diet (H). Blood cholesterol levels at week 16 in male offspring under normal diet (I), HFSS diet (J), as well as female offspring under normal diet (K) and HFSS diet (L). Blood CRP levels at week 16 in male offspring under normal diet (M), HFSS diet (N), as well as female offspring under normal diet (O) and HFSS diet (P). Statistical analysis was conducted using one-way ANOVA, followed by post-hoc Tukey's multiple comparisons test. Significance levels are denoted as *P < 0.05, **P < 0.01. Each group comprised n = 17-28 animals.

**Figure 5:**
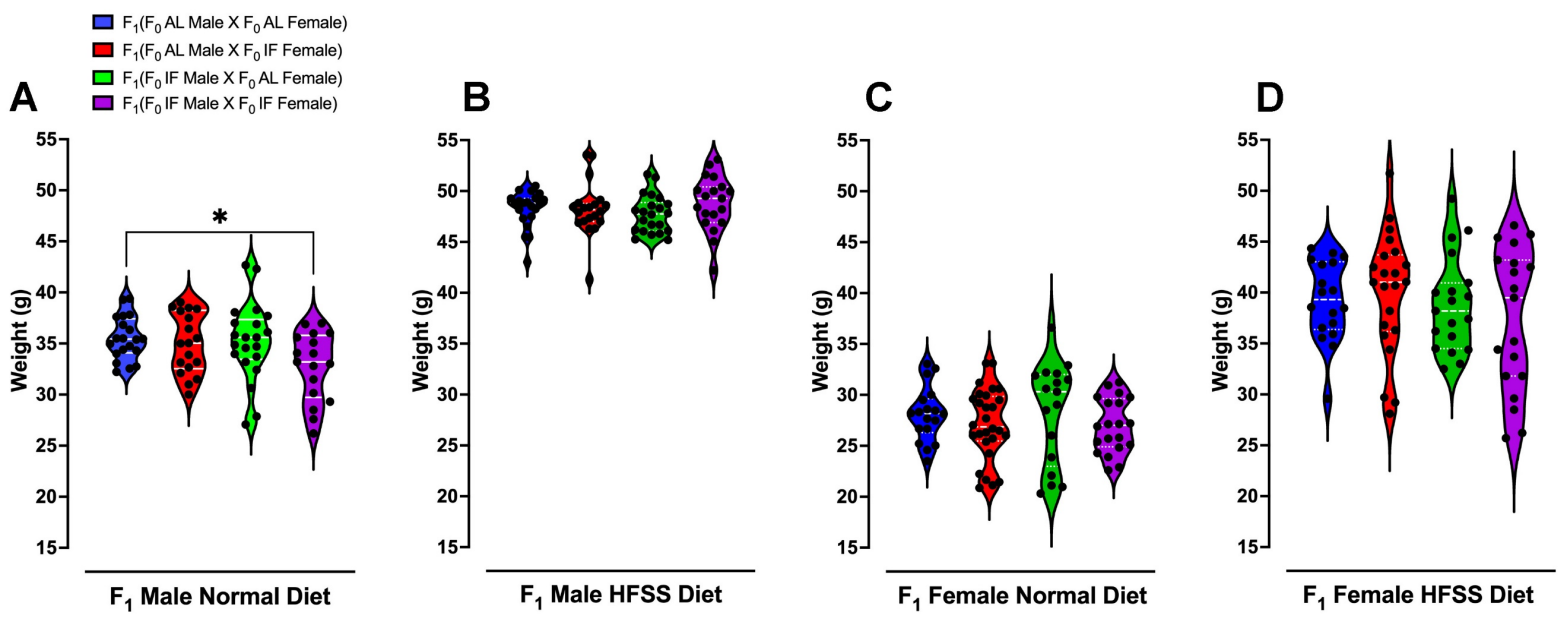
** Body Weight of Offspring Animals**. Violin plots illustrate the body weight distributions of offspring normal diet male (A), HFSS diet male (B), normal diet female (C) and HFSS diet female (D) animals at 16 weeks after weaning. Statistical analysis was performed using one-way ANOVA, followed by post-hoc Šídák's multiple comparisons test. Significance levels are denoted as *P < 0.05 compared to F_1_ (F_0_ AL male X F_0_ AL Female) animals. Each group consisted of n = 17-28 animals.

All analyses were performed using GraphPad Prism version 10.0. For **Figure 2** and **Supplementary Figure 1** (F_0_ generation), as well as **Supplementary Figure 7** (F_1_ generation), body weight and changes were presented as means ± standard deviation (s.d.). For all comparisons involving multiple time points in **Figure 2** (F_0_ generation) and **Supplementary Figure 2** (F_0_ generation), a two-way repeated-measurements analysis of variance (ANOVA), followed by post-hoc Šídák's multiple comparisons test, was conducted to determine P values compared to control groups. For **Figure 3** (F_1_ generation), in the analysis of the glucose and insulin levels following the glucose tolerance test, two methods were employed. Firstly, the XY graph depicts individual replicates with means connected, and a two-way repeated-measurements ANOVA, followed by post-hoc uncorrected Fisher's LSD comparisons test, was utilized to determine P values compared to respective time-specific control groups. Secondly, the Area Under the Curve analysis was performed using one-way ANOVA, followed by Tukey's multiple comparisons test, to determine P values. Data were presented as means ± standard deviation (s.d.). For **Figures 4** and **5** data were displayed using violin plots, and a one-way ANOVA, followed by Tukey's multiple comparisons test, was used to determine P values compared to respective time-specific control groups. **Supplementary Figures 3-6** (F_1_ generation), data were presented as means ± standard deviation (s.d.). A two-way repeated-measurements ANOVA was conducted, followed by Tukey's multiple comparisons test, to determine P values.

## Results

### Effects of intermittent fasting on parental animals and the mating process

Two cohorts comprising 128 parental C57BL/6 mice, and 319 offspring from four mating combinations across 16 groups, were placed on normal or HFSS dietary regimes over four months to obtain data for this study. Parental male and female C57BL/6 mice were all initially fed a normal diet on a caloric basis until the start of the dietary intervention. At 6 weeks of age, mice were randomly assigned to either *ad libitum* (AL) feeding or daily 16-hour fasting (IF) schedule (**Fig. 1**). Parental mice on AL feeding exhibited significantly greater body weight changes (**Fig. 2A and B; Supplementary Fig. 1A and B**) than those on the IF, regardless of sex, during the 16-week dietary intervention period.

To evaluate the impact of IF on energy metabolism in parental animals, we quantified blood glucose, ketone, cholesterol, and the inflammation marker C-reactive protein (CRP) levels at baseline and at 8 and 16 weeks of dietary intervention (**Fig. 2C - J**). Male and female mice on IF exhibited significantly lower glucose levels (**Fig. 2C and D**), significantly higher ketone levels (**Fig. 2E and F**) and significantly decreased HbA1c levels (**Supplementary Fig. 2A and B**) compared to the AL control mice. Furthermore, cholesterol levels were significantly reduced in IF mice from both sexes at week 16 (**Fig. 2G and H**). CRP levels were significantly reduced by IF in female mice at 16 weeks (**Fig. 2I and J**). However, no differences were observed in triglyceride levels between IF and AL animals.

After 16 weeks of dietary intervention, parental male and female mice from both groups were paired for mating (**Fig. 1**). During the mating period, all mice had *ad libitum* access to food and water. Upon confirmation of pregnancy, male mice were separated from the cages, and female mice had unrestricted access to food and water throughout pregnancy and a subsequent 4-week period. When they were 4 weeks old the offspring from all four parental groups, namely F_1_(F_0_ AL Male X F_0_ AL Female); F_1_(F_0_ AL Male X F_0_ IF Female); F_1_(F_0_ IF Male X F_0_ AL Female); and F_1_(F_0_ IF Male X F_0_ IF Female), were separated from their mothers and assigned to either the usual normal diet or a high-fat, high-sugar, and high-salt (HFSS) diet (**Fig. 1**).

### Physiological and metabolic phenotypes of offspring

The offspring were then maintained on either a normal or HFSS diet for an additional 16 weeks (**Fig. 1**). Throughout this period, they underwent evaluations for physiological changes and metabolic phenotypes to investigate the potential effects of maternal and/or paternal IF (**Figs. 3 - 5**). We measured blood glucose and insulin levels in all groups of offspring after an intraperitoneal glucose tolerance challenge (**Fig. 3**). This glucose tolerance test assesses how effectively the body clears a glucose load. For males on the normal diet, those from an AL female and IF male crossing exhibited a significantly greater glucose area under the curve (AUC) compared to offspring from AL female/AL male crossings (**Fig. 3A and B**). When on the normal diet males from IF female/IF male crossings exhibited a lower AUC compared to males from AL female/AL male crossings (**Fig. 3A and B**). However, no significant differences were observed in blood glucose AUC levels among female offspring on the normal diet between any of the four different combinations of parental groups (**Fig. 3C and D**). For male offspring on the HFSS diet, there were no significant differences in glucose AUC among between any of the four parental diet groups (**Fig. 3E and F**). However, female offspring on the HFSS diet, from IF male/IF female crossings exhibited significantly lower glucose AUC levels compared to offspring from each of the other three groups (**Fig. 3G and H**). Some variations in fasting glucose and insulin levels were observed between the normal and HFSS-treated offspring. However, these differences did not affect our analyzes, as all comparisons were conducted within each group and analyzed separately (**Fig. 3**).

We next measured plasma insulin levels at baseline (0 minutes; before peritoneal glucose injection) and at 30- and 120- minutes thereafter. For mice on the normal diet, insulin AUC analysis revealed significantly elevated insulin levels in male offspring of male IF and female IF crossings compared to males from AL/AL or male AL/female IF crossings (**Fig. 3I and J**). For offspring females on the normal diet those from IF male/IF female crossings exhibited elevated insulin AUC levels compared to female offspring from each of the other three groups (**Fig. 3K and L**). For offspring males on the HFSS diet, there were no differences in insulin AUC levels among the four groups (**Fig. 3M and N**). For offspring females on the HFSS diet those from IF male/IF female crossings exhibited an elevated insulin AUC level compared to those from AL male/AL female crossings (**Fig. 3O and P**). For offspring on the normal diet, the insulin levels obtained just before the intraperitoneal glucose injection were significantly higher in male and female offspring from IF male/IF female crossings compared to offspring from AL male/AL female crossings (**Fig. 3I and J**).

Plasma glucose, ketone, cholesterol, triglyceride, and CRP levels were measured immediately following weaning (Week 0), and at 12 and 16 weeks of dietary intervention preceding the glucose tolerance test (**Fig. 4 A-P and Supplementary Figs. 3-5**). Offspring males and females from IF male/IF female crossings had lower plasma glucose levels compared to those from AL male/AL female crossings (**Supplementary Fig. 3A and C**). Offspring females from IF male/IF female crossings exhibited significantly higher ketone levels compared to offspring females from AL male/AL female crossings (**Supplementary Fig. 3G**). Notably, at 16 weeks following the HFSS diet, female offspring from both IF parents displayed significantly lower glucose levels and higher ketone levels (**Fig. 3 D and H**). Furthermore, male offspring on the normal diet from parents that had both been on IF exhibited a significant reduction in body weight from week 10 onwards compared to offspring from parents that had both been on AL (**Fig. 5A and Supplementary Fig. 7**).

## Discussion

Parental nutritional status can significantly impact offspring through epigenetic inheritance mechanisms 17-22. Previous research has explored the phenotypic outcomes in offspring resulting from parental obesogenic diets 22, 23, the possible effects of parental IF on offspring metabolic traits are less known. Recent studies have investigated the effects of maternal IF on the epigenetic landscape and metabolic phenotypes of offspring, exploring potential mechanisms of inheritance through epigenetic modifications such as DNA methylation 12-15. However, the impact of combined or individual parental IF on offspring metabolic phenotypes remains poorly understood. The present study begins to fill this knowledge gap. Our study examines the effects of various combinations of parental IF on offspring metabolic traits. Specifically, we explored the impact of both parents following either an AL or IF diet, as well as the effects of maternal or paternal IF alone, on the metabolic outcomes in offspring. We found that compared to offspring from parents fed AL, those born to parents that had both been on IF exhibit differences in markers of energy metabolism and that vary depending upon the sex of the offspring. Offspring females (but not offspring males) from IF/IF parents exhibited improved glucose tolerance when on a HFSS diet but not when on the normal diet. No significant differences in body weight were observed between offspring groups within diet- and sex-specific categories (e.g., normal diet male, normal diet female, HFSS diet male, and HFSS diet female). The only notable difference in body weight was observed between offspring of AL parents and those of IF parents within the normal diet group. Therefore, the body weights are unlikely to confound the comparison of glucose and insulin AUC values.

Our findings suggest that parental fasting leads to enhanced insulin production both at basal levels (minute 0 in both female and male offspring animals under normal diet) and following glucose injection, indicating that these offspring inherit epigenetic mechanisms that may be responsible for the observed increase in insulin secretion. While we observed no metabolic benefits from the HFSS diet in male offspring from IF parental animals, male offspring from IF parents on a normal diet exhibited increased plasma insulin levels and reduced body weight compared to those from AL parents. This may suggest that male offspring may inherit certain metabolic traits from IF parents. Sex-specific transgenerational effects of parental diet have been reported by multiple studies across different species 24-30. These studies have demonstrated significant differences in transgenerational responses to ancestral diet, with variation observed between genotypes and sexes across both the first and second descendant generations 24, 25.

In conclusion, our study represents a novel exploration into how parental IF may confer metabolic benefits to offspring, likely mediated by epigenetic mechanisms inherited from both parents. Notably, female offspring of IF parents, but not males, retained these metabolic advantages even when exposed to an HFSS diet. The effects of IF on age-related diseases and cognitive decline have been extensively studied, including in our own research, which has established that IF offers protection against cardiovascular and neurodegenerative diseases 2, 4, 10, 31-36. While our study addresses this important gap, several limitations need to be addressed in future research. These include identifying the precise epigenetic mechanisms responsible for the metabolic differences observed between offspring of AL and IF parents, as well as investigating how hormonal and physiological differences may contribute to the differential metabolic responses observed in female versus male offspring, particularly under HFSS diet conditions. Although our results offer valuable insights, it's crucial to acknowledge the inherent metabolic disparities between species, raising caution in directly translating our observations to humans.

## Supplementary Material

Supplementary figures and data legends.

Supplementary source data for figure 2.

Supplementary source data for figure 3.

Supplementary source data for figure 4.

Supplementary source data for figure 5.

Supplementary source data for supplementary figure 1.

Supplementary source data for supplementary figure 2.

Supplementary source data for supplementary figure 3.

Supplementary source data for supplementary figure 4.

Supplementary source data for supplementary figure 5.

Supplementary source data for supplementary figure 6.

Supplementary source data for supplementary figure 7.
