# Supplementary material for: Impact of Parental Time-Restricted Feeding on Offspring Metabolic Phenotypic Traits
Source: Int J Biol Sci. 2025 Feb 10;21(5):1852–62. doi: 10.7150/ijbs.107469 (PMC11900813; doi:10.7150/ijbs.107469)
Supplement: Supplementary file 1 — Supplementary figures and data legends. [file ijbsv21p1852s1.pdf]

Impact of Parental Time-Restricted Feeding on Offspring Metabolic Phenotypic Traits

Yibo Fan[a,b], Xiangyuan Peng[a,b], Nishat I. Tabassum[a,b], Xiangru Cheng[a,b], Sharmelee Selvaraji[c], Vivian Tran[a,b], Tayla A. Gibson Hughes[a,b], Buddhila Wickramasinghe[a,b], Abdulsatar Jamal[a,b], Quynh Nhu Dinh[a,b], Mathias Gelderblom[d], Grant R. Drummond[a,b], Christopher G. Sobey[a,b], Jim Penman[e], Terrance G. Johns[e], Raghu Vemuganti[f], Jayantha Gunaratne[g,h], Mark P. Mattson[i], Dong-Gyu Jo[j], Maria Jelinic[a,b,1], Thiruma V. Arumugam[a,b,j,1]

[a]Centre for Cardiovascular Biology and Disease Research, La Trobe Institute for Molecular Science, La Trobe University, Melbourne, Australia.
[b]Department of Microbiology, Anatomy, Physiology and Pharmacology, School of Agriculture, Biomedicine and Environment, La Trobe University, Melbourne, Australia.
[c]Research Laboratory of Electronics, Department of Materials Science and Engineering, Massachusetts Institute of Technology, Boston, MA, USA.
[d]Department of Neurology, University Medical Center Hamburg-Eppendorf, Hamburg, Germany.
[e]Epigenes Australia Pty Ltd., Melbourne, Victoria, Australia.
[f]Department of Neurological Surgery, University of Wisconsin, Madison, WI, USA.
[g]Translational Biomedical Proteomics Laboratory, Institute of Molecular and Cell Biology, Agency for Science, Technology and Research, Singapore, Singapore.
[h]Department of Anatomy, Yong Loo Lin School of Medicine, National University of Singapore, Singapore, Singapore.
[i]Department of Neuroscience, Johns Hopkins University School of Medicine, Baltimore, MD, USA.
[j]School of Pharmacy, Sungkyunkwan University, Suwon, Republic of Korea.

Corresponding authors: Thiruma V. Arumugam or Maria Jelinic**,** Centre for Cardiovascular Biology and Disease Research, La Trobe Institute for Molecular Science and Department of Microbiology, Anatomy, Physiology and Pharmacology, School of Agriculture, Biomedicine and Environment, La Trobe University, Bundoora, VIC, Australia. **E-mail:** g.arumugam@latrobe.edu.au; garriearumugam@gmail.com; or M.Jelinic@latrobe.edu.au

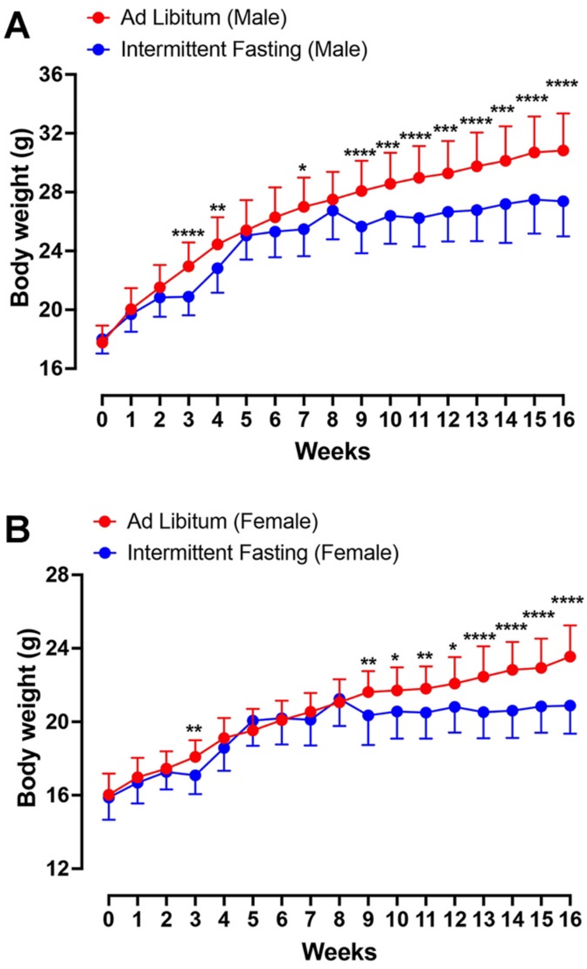

Supplementary Figure 1: Impact of Intermittent Fasting on Body Weight of Parental Animals. The figure illustrates the body weight trajectories of male (A) and female (B) parental animals following daily 16-hour intermittent fasting (IF) over a 16-week period. Data are presented as means ± standard deviation. Statistical analysis was conducted using two-way repeated-measure ANOVA, followed by post-hoc Šídák's multiple comparisons test. Significance levels are indicated as *P < 0.05, **P < 0.01, ***P < 0.001, ****P < 0.0001 compared to IF animals. Each group consisted of n = 32 animals.

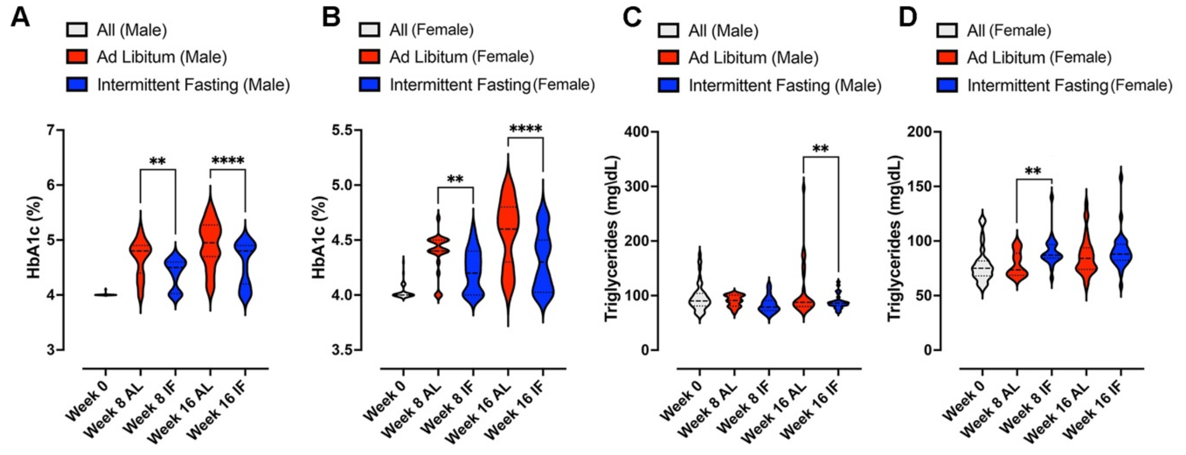

Supplementary Figure 2: Impact of Intermittent Fasting on HbA1c and Triglyceride Levels of Parental Animals. Violin plots depict blood HbA1c levels in male (A) and female (B) parental animals, as well as triglyceride levels in male (C) and female (D) parental animals. Statistical analysis was conducted using two-way ANOVA, followed by post-hoc Šídák's multiple comparisons test. Significance levels are denoted as **P < 0.001, ****P < 0.0001 compared to *ad libitum* (AL) animals. Each group consisted of n = 32 animals.

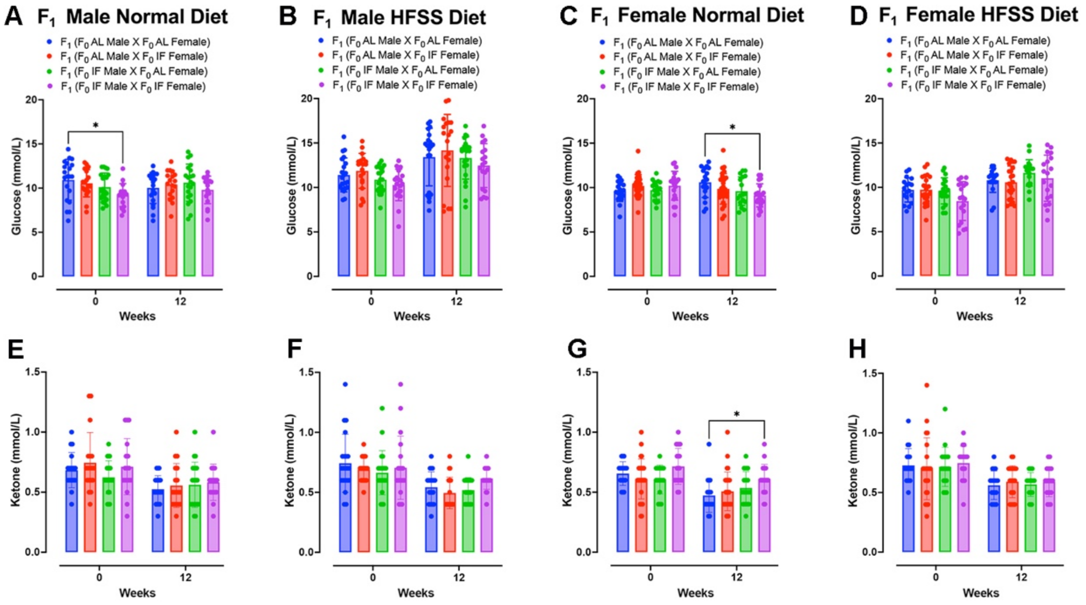

Supplementary Figure 3: Blood Glucose and Ketone Levels of Offspring Animals. Bar graphs illustrate the blood glucose and ketone levels at week 0 (immediately after weaning) and week 12 in male offspring under normal diet (A and E), HFSS diet (B and F), as well as female offspring under normal diet (C and G) and HFSS diet (D and H). Data are displayed as means ± standard deviation. Statistical analysis was conducted using one-way ANOVA, followed by post-hoc Tukey's multiple comparisons test. Significance levels are denoted as *P < 0.05. Each group comprised n = 17-28 animals.

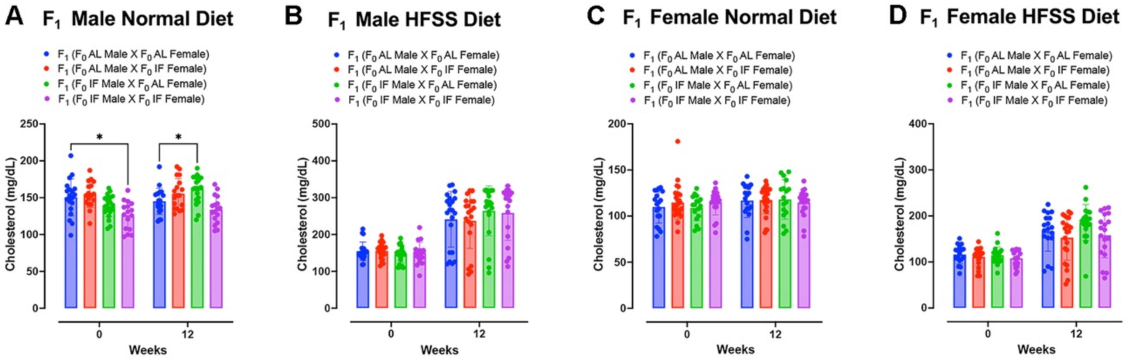

Supplementary Figure 4: Blood Cholesterol Levels of Offspring Animals. Bar graphs depict the blood cholesterol levels at week 0 (immediately after weaning) and week 12 in male offspring under normal diet (A), HFSS diet (B), as well as female offspring under normal diet (C) and HFSS diet (D). Data are displayed as means ± standard deviation. Statistical analysis was performed using one-way ANOVA, followed by post-hoc Tukey's multiple comparisons test. Significance levels are indicated as *P < 0.05. Each group consisted of n = 17-28 animals.

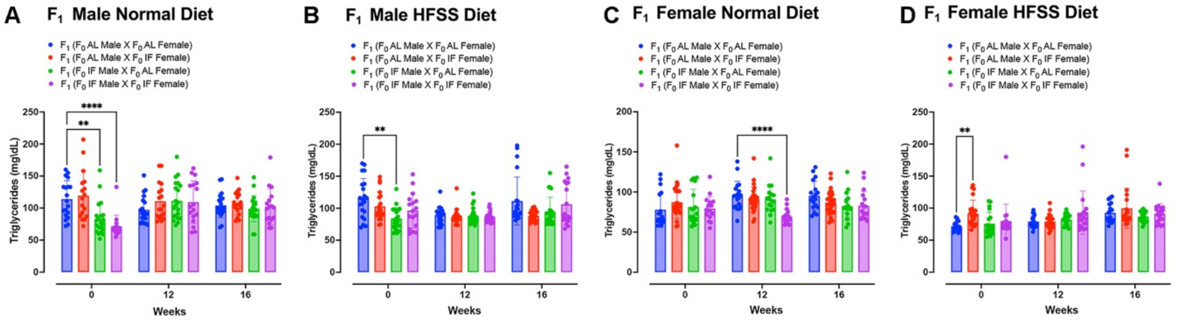

Supplementary Figure 5: Blood Triglyceride Levels of Offspring Animals. Bar graphs depict the blood triglyceride levels at week 0 (immediately after weaning) and week 12 in male offspring under normal diet (A), HFSS diet (B), as well as female offspring under normal diet (C) and HFSS diet (D). Data are displayed as means ± standard deviation. Statistical analysis was performed using one-way ANOVA, followed by post-hoc Tukey's multiple comparisons test. Significance levels are indicated as **P < 0.01, ****P<0.0001. Each group consisted of n = 17-28 animals.

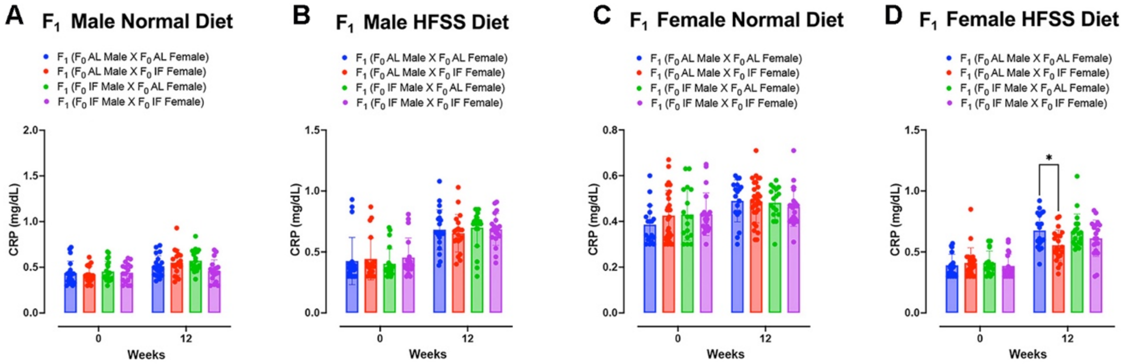

Supplementary Figure 6: Blood CRP Levels of Offspring Animals. Bar graphs illustrate the blood CRP levels at week 0 (immediately after weaning) and week 12 in male offspring under normal diet (A), HFSS diet (B), as well as female offspring under normal diet (C) and HFSS diet (D). Data are displayed as means ± standard deviation. Statistical analysis was conducted using one-way ANOVA, followed by post-hoc Tukey's multiple comparisons test. Significance levels are denoted as *P < 0.05. Each group comprised n = 17-28 animals.

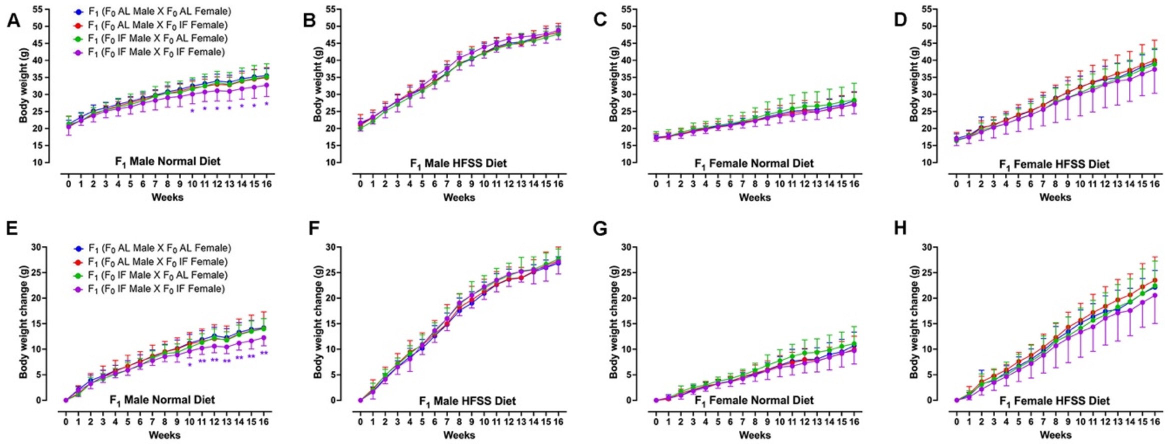

Supplementary Figure 7: Body Weight of Offspring Animals. The figure presents the body weight trajectories of male offspring (A, B, E and F) and female offspring (C, D, G and H) under normal and HFSS diets over 16-week periods. Data are displayed as means ± standard deviation. Statistical analysis was conducted using two-way repeated-measurement ANOVA, followed by post-hoc Šídák's multiple comparisons test. Significance levels are indicated as *P < 0.05, **P < 0.01 compared to F_1 (F_0 AL male X F_0 AL Female) animals. Each group comprised n = 17-28 animals.

Source data files

Source data for Figure 2: Raw data for Figure 2, Impact of Intermittent Fasting on Parental Animals.

Source data for Figure 3: Raw data for Figure 3, Glucose and Insulin Response in Offspring Following Glucose Tolerance Test.

Source data for Figure 4: Raw data for Figure 4, Blood Glucose, Ketone, Cholesterol and CRP Levels of Offspring Animals.

Source data for Figure 5: Raw data for Figure 5, Body Weight of Offspring Animals.

Source data for Supplement Figure 1: Raw data for supplementary Figure 1.

Source data for Supplement Figure 2: Raw data for supplementary Figure 2.

Source data for Supplement Figure 3: Raw data for supplementary Figure 3.

Source data for Supplement Figure 4: Raw data for supplementary Figure 4.

Source data for Supplement Figure 5: Raw data for supplementary Figure 5.

Source data for Supplement Figure 6: Raw data for supplementary Figure 6.

Source data for Supplement Figure 7: Raw data for supplementary Figure 7.
